# Supplementary material for: Distribution Patterns of Gymnosperm Species along Elevations on the Qinghai–Tibet Plateau: Effects of Climatic Seasonality, Energy–Water, and Physical Tolerance Variables
Source: Plants (Basel). 2023 Dec 4;12(23):4066. doi: 10.3390/plants12234066 (PMC10708526; doi:10.3390/plants12234066)
Supplement: Supplementary file 1 [file plants-12-04066-s001.zip › Supplementary figure.pdf]

Distribution patterns of gymnosperm species along elevations on the Qinghai–Tibet Plateau: Effects of climatic seasonality, energy–water, and physical tolerance variables

Muhammad Umair[1,2*], Xiaofei Hu[1], Qi Cheng[1], Shahzad Ali[1], Jian Ni[1*]

[1]College of Life Sciences, Zhejiang Normal University, Jinhua 321004, China

[2]School of Agriculture and Biology, Shanghai Jiao Tong University, Shanghai 200240, China

Correspondence: umairm@zjnu.edu.cn; nijian@zjnu.edu.cn

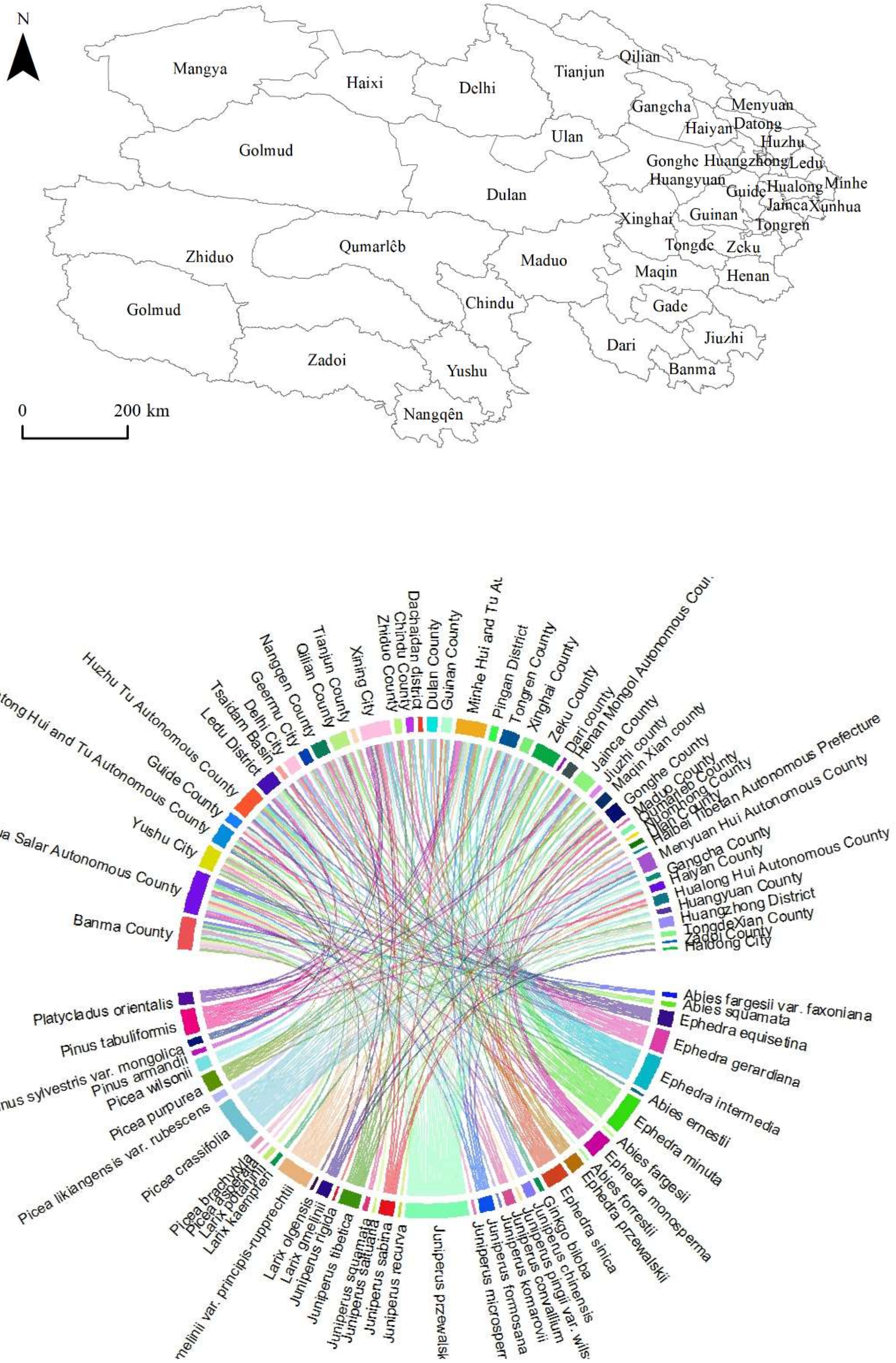

Figure S1. Regional distribution of gymnosperm species in the Qinghai–Tibet Plateau, China.

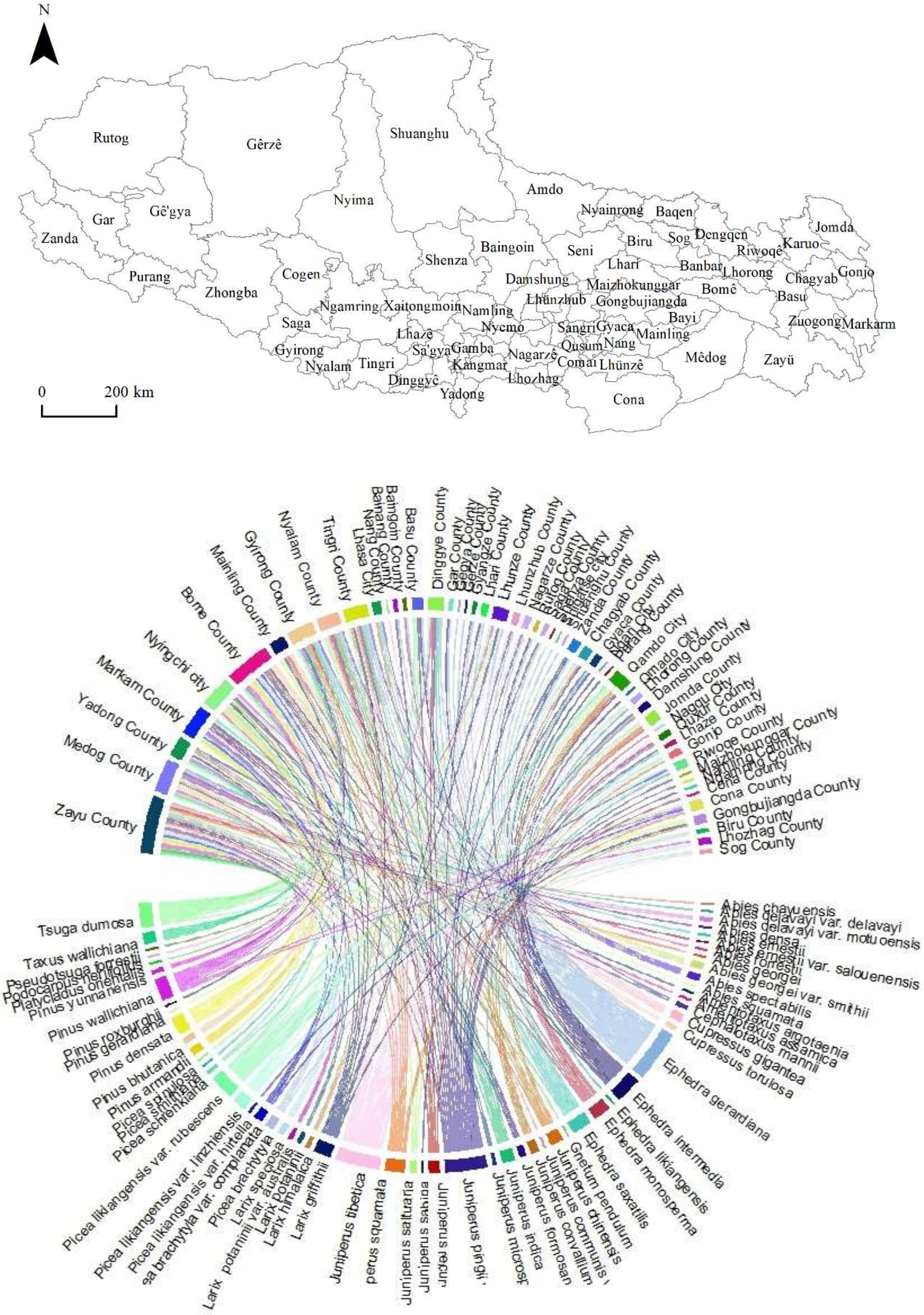

Figure S2. Regional distribution of gymnosperm species in the Qinghai–Tibet Plateau, China.

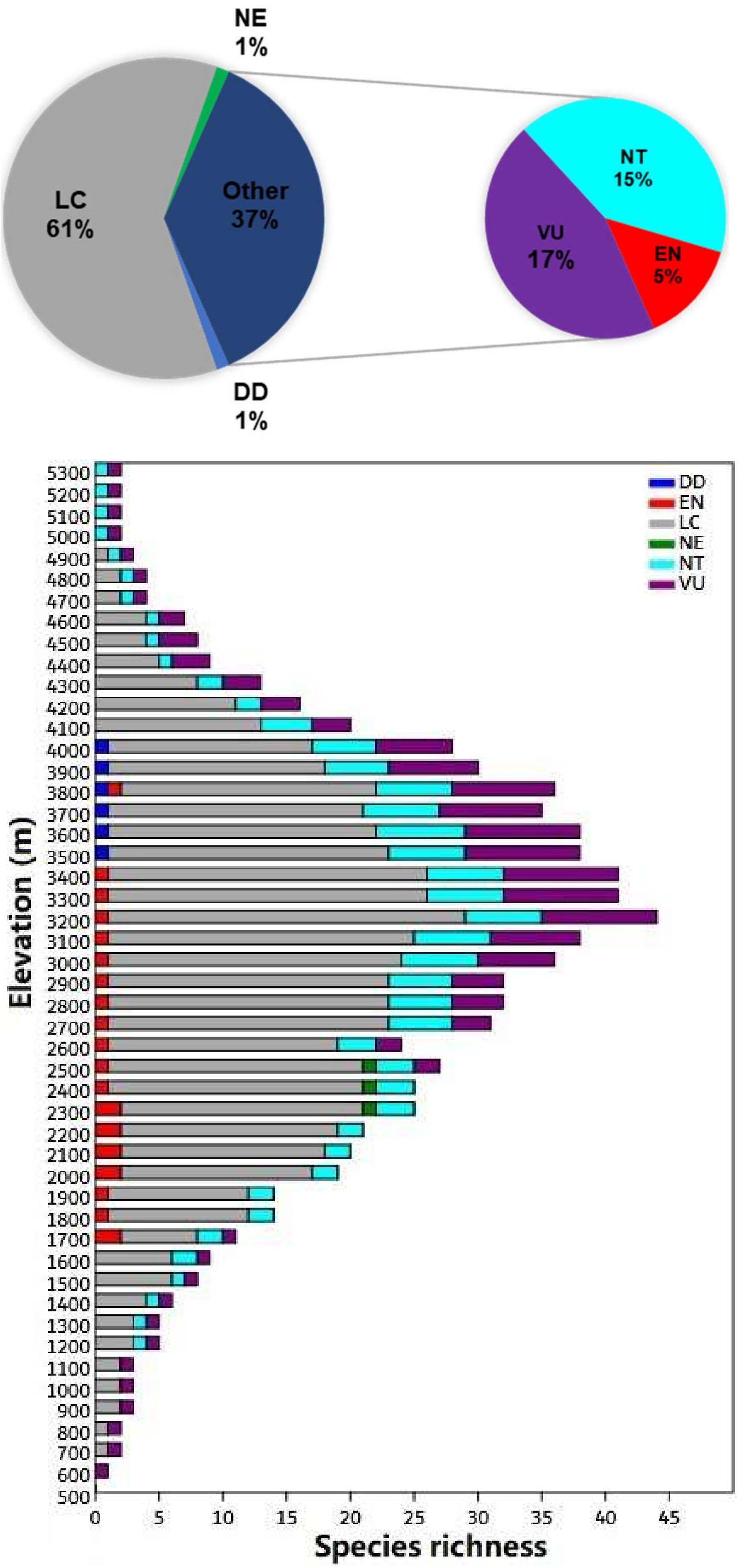

Figure S3. The conservation status of gymnosperm species along the elevations on the Qinghai–Tibetan Plateau, China.

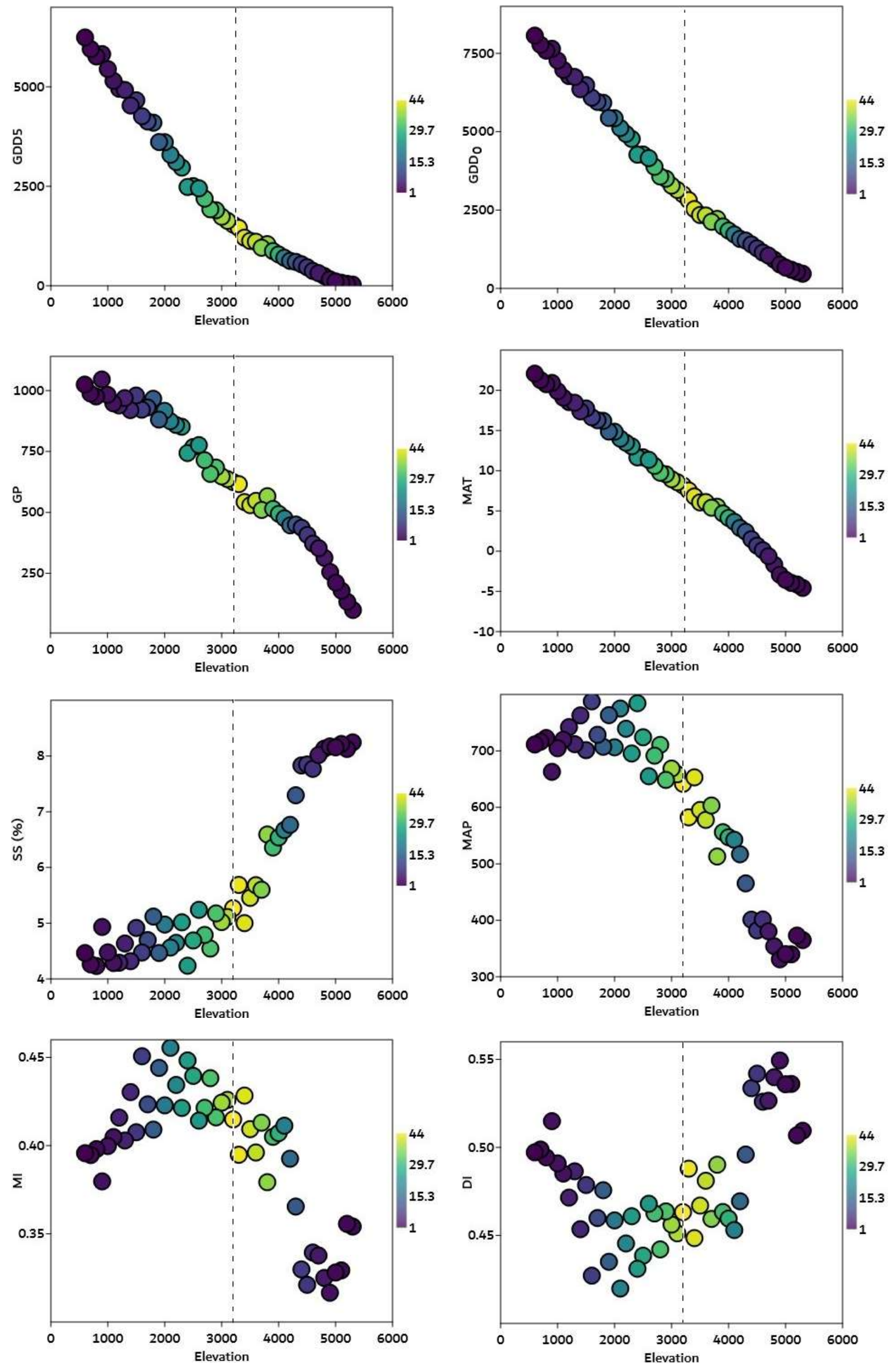

Figure S4. Scatter plots showing the relationship between elevation (m) and energy-water variables. The scale indicates gymnosperm richness along elevation gradients in the Qinghai–Tibet Plateau, China.

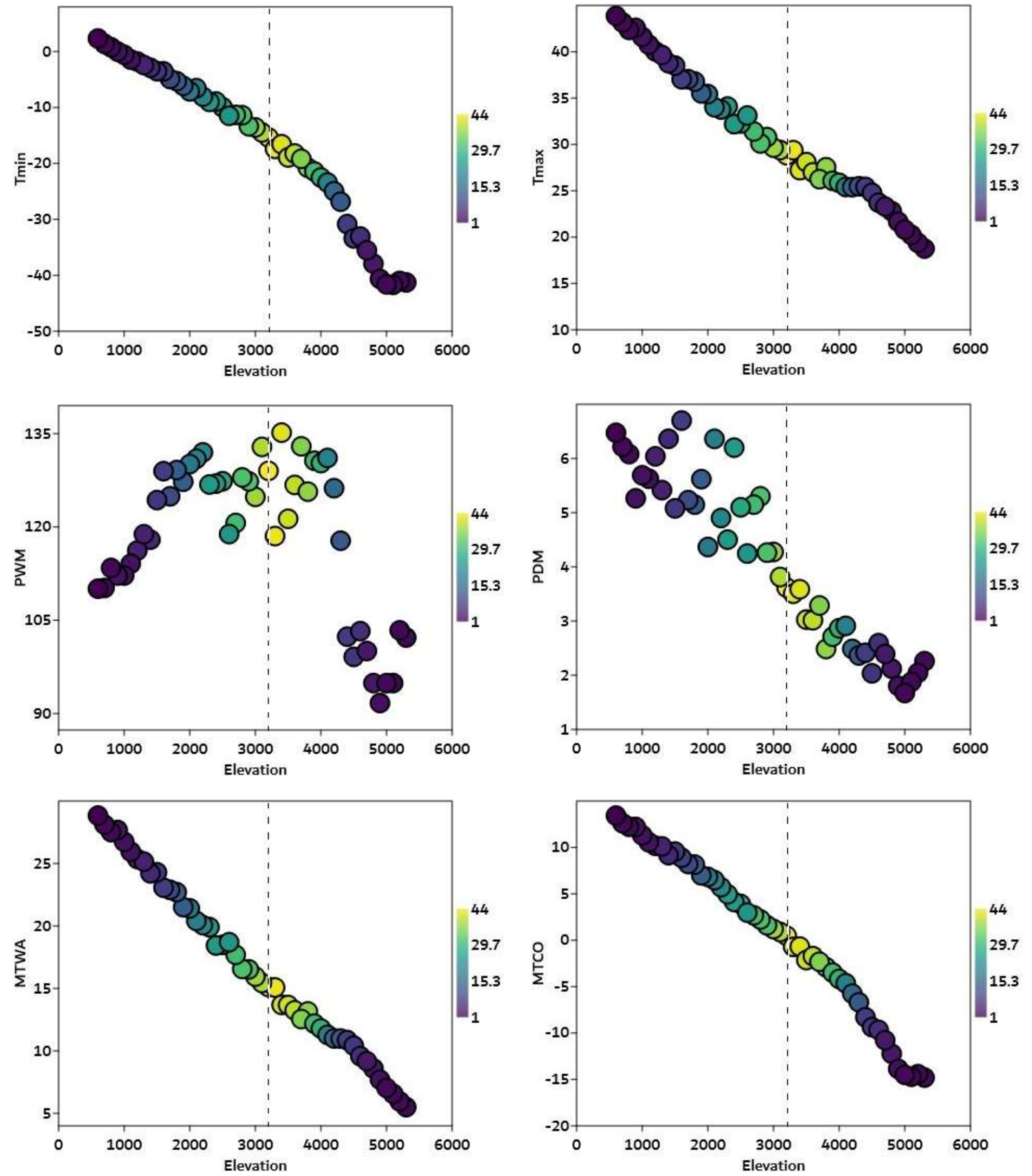

Figure S5. Scatter plots showing the relationship between elevation (m) and physical tolerance variables. The scale indicates gymnosperm richness along elevation gradients in the Qingha–Tibet Plateau, China.

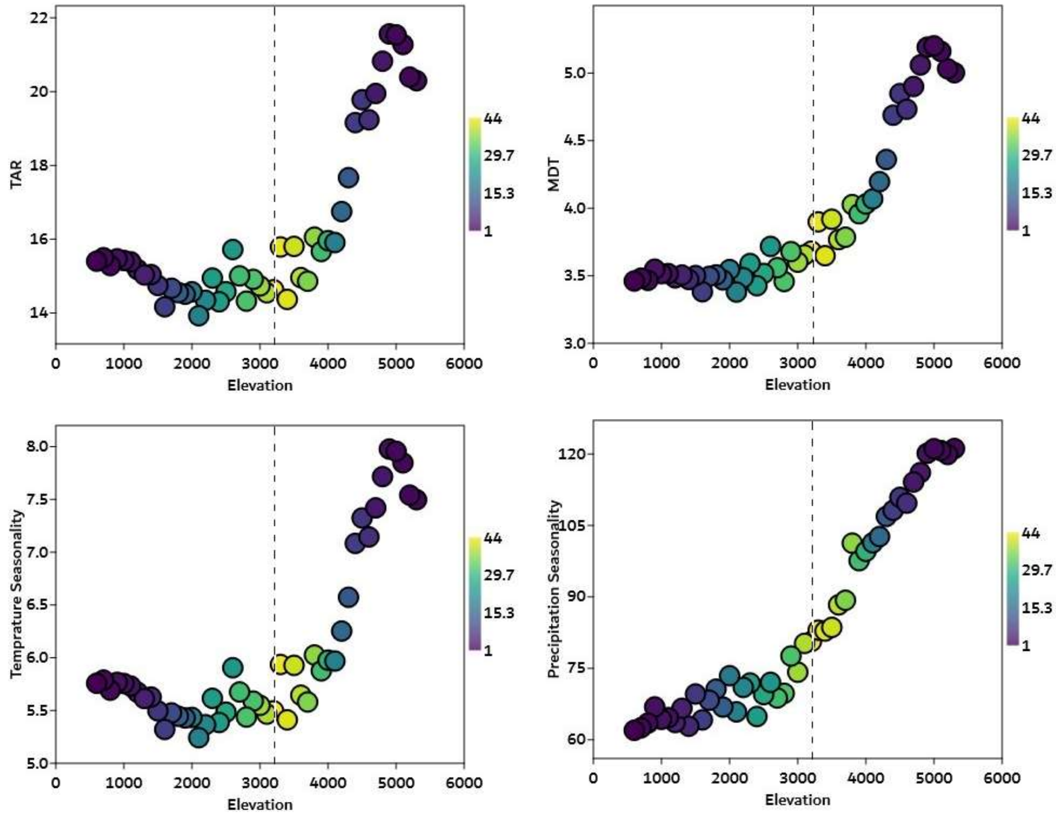

Figure S6. Scatter plots showing the relationship between elevation (m) and climatic seasonality variables. The scale indicates gymnosperm richness along elevation gradients in the Qinghai–Tibet Plateau, China.

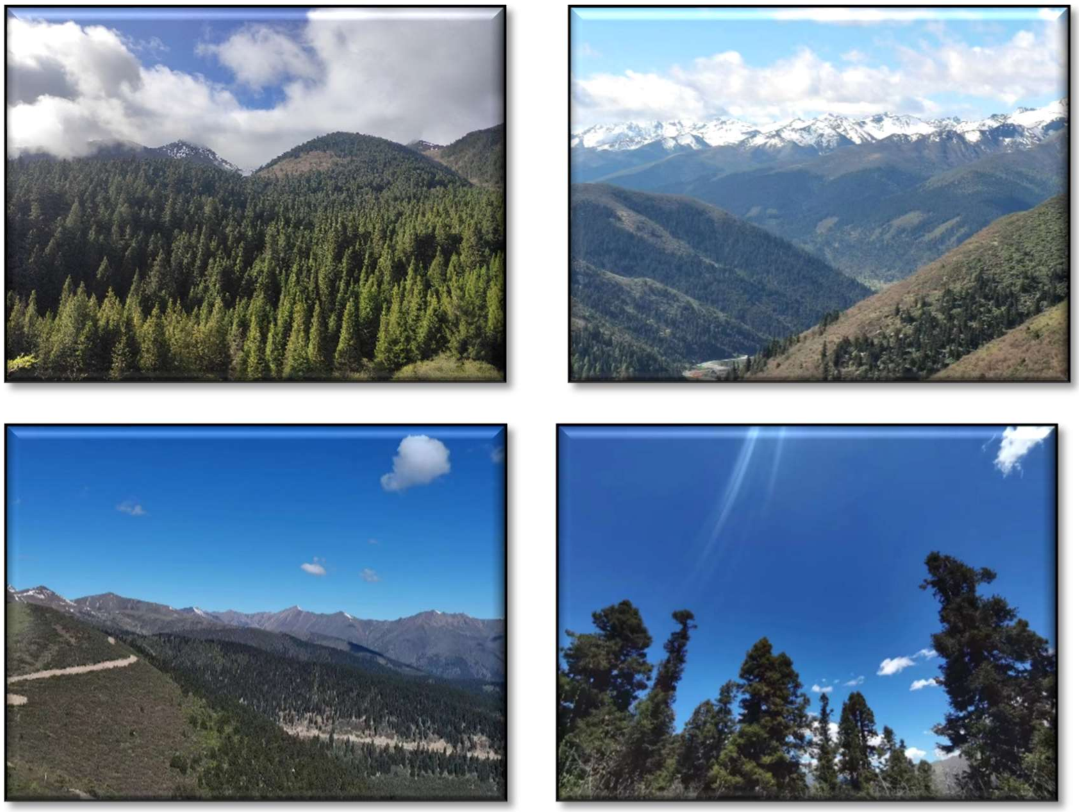

Figure S7. Spruce forest distribution in the QTP's sub-alpine zone (3250 m a.s.l.) between 32°30′-32°54′ north latitude and 103°25′-104°45′ east longitude.
