# Supplementary material for: Distribution Patterns of Gymnosperm Species along Elevations on the Qinghai–Tibet Plateau: Effects of Climatic Seasonality, Energy–Water, and Physical Tolerance Variables
Source: Plants (Basel). 2023 Dec 4;12(23):4066. doi: 10.3390/plants12234066 (PMC10708526; doi:10.3390/plants12234066)
Supplement: Supplementary file 1 [file plants-12-04066-s001.zip › Supplementary Table.pdf]

Distribution patterns of gymnosperm species along elevations on the Qinghai–Tibet Plateau: Effects of climatic seasonality, energy–water, and physical tolerance variables

Muhammad Umair[1,2*], Xiaofei Hu[1], Qi Cheng[1], Shahzad Ali[1], Jian Ni[1*]

[1]College of Life Sciences, Zhejiang Normal University, Jinhua 321004, China

[2]School of Agriculture and Biology, Shanghai Jiao Tong University, Shanghai 200240, China

Correspondence: umairm@zjnu.edu.cn; nijian@zjnu.edu.cn

Table S1. Native distribution range of selected gymnosperm species.

Sr. No.	Species name	Native distribution
1	*Cupressus gigantea*	China South-Central, Tibet
2	*Cupressus torulosa*	Nepal, Pakistan, Tibet, West Himalaya
3	*Juniperus formosana*	China Southeast, Qinghai, Tibet, Taiwan
4	*Juniperus recurva*	Assam, China South-Central, East Himalaya, Myanmar, Nepal, Qinghai, Tibet, West Himalaya
5	*Juniperus rigida*	China North-Central, Inner Mongolia, Japan, Korea, Manchuria, Nansei-shoto, Primorye, Qinghai, Sakhalin
6	*Juniperus communis var. saxatilis*	Afghanistan, Albania, Altay, Amur, British Columbia, Bulgaria, Buryatiya, California, Chita, Corse, Cyprus, Czechoslovakia, East Aegean Is., East European Russia, Finland, France, Føroyar, Germany, Greece, Greenland, Iceland, Inner Mongolia, Iran, Iraq, Irkutsk, Italy, Japan, Kamchatka, Kazakhstan, Khabarovsk, Kirgizstan, Korea, Krasnoyarsk, Kuril Is., Lebanon-Syria, Magadan, Manchuria, Mongolia, Nepal, North Caucasus, Norway, Nova Scotia, Oregon, Pakistan, Poland, Primorye, Romania, Sakhalin, Spain, Sweden, Switzerland, Tadzhikistan, Transcaucasus, Turkey, Turkey-in-Europe, Turkmenistan, Tuva, Ukraine, Uzbekistan, Washington, West Himalaya, West Siberia, Xinjiang, Yakutskiya, Yugoslavia, Yukon
7	*Platycladus orientalis*	Amur, China North-Central, China Southeast, Qinghai, Tibet, Khabarovsk, Korea
8	*Juniperus chinensis*	China North-Central, China South-Central, China Southeast, Qinghai, Tibet, Inner Mongolia, Japan, Khabarovsk, Korea, Kuril Is., Manchuria, Myanmar, Primorye, Sakhalin, Taiwan
9	*Juniperus convallium*	China North-Central, China South-Central, Qinghai, Tibet
10	*Juniperus komarovii*	China North-Central, China South-Central, Qinghai
11	*Juniperus microsperma*	China South-Central, Qinghai, Tibet
12	*Juniperus pingii var. wilsonii*	China North-Central, China South-Central, Qinghai, Tibet
13	*Juniperus przewalskii*	China North-Central, China South-Central, Inner Mongolia, Qinghai
14	*Juniperus saltuaria*	China North-Central, China South-Central, Qinghai, Tibet
15	*Juniperus squamata*	Afghanistan, Assam, China North-Central, China South-Central, China Southeast, East Himalaya, Myanmar, Nepal, Pakistan, Qinghai, Tibet, West Himalaya
16	*Juniperus tibetica*	China North-Central, China South-Central, Qinghai, Tibet
17	*Juniperus sabina*	Albania, Algeria, Altay, Amur, Austria, Bulgaria, China North-Central, Chita, Czechoslovakia, East European Russia, France, Germany, Greece, Inner Mongolia, Italy, Kazakhstan, Khabarovsk, Kirgizstan, Korea, Krym, Manchuria, Mongolia, North Caucasus, Poland, Primorye, Qinghai, Tibet, South European Russi, Spain, Switzerland, Tadzhikistan, Transcaucasus, Turkey, Tuva, Ukraine, West Siberia, Xinjiang, Yugoslavia
18	*Juniperus indica*	China South-Central, East Himalaya, Nepal, Pakistan, Tibet, West Himalaya

19	*Ephedra equisetina*	Altay, China North-Central, Inner Mongolia, Kirgizstan, Krasnoyarsk, Mongolia, North Caucasus, Primorye, Qinghai, Tadzhikistan, Turkey, Turkmenistan, Tuva, Uzbekistan, West Siberia, Xinjiang
20	*Ephedra gerardiana*	Afghanistan, Altay, East Himalaya, Nepal, Pakistan, Qinghai, Tadzhikistan, Tibet, Tuva, West Himalaya, Xinjiang
21	*Ephedra intermedia*	Afghanistan, Altay, Inner Mongolia, Iran, Kazakhstan, Kirgizstan, Mongolia, Pakistan, Tadzhikistan, Qinghai, Tibet, Transcaucasus, Turkmenistan, Uzbekistan, West Himalaya, West Siberia, Xinjiang
22	*Ephedra likiangensis*	China South-Central, Tibet
23	*Ephedra saxatilis*	China South-Central, East Himalaya, Nepal, Tibet
24	*Ephedra minuta*	China South-Central, Qinghai, Tajikistan
25	*Ephedra monosperma*	Buryatiya, China North-Central, China South-Central, Chita, Inner Mongolia, Irkutsk, Krasnoyarsk, Mongolia, Primorye, Qinghai, Tibet, Tuva, Xinjiang, Yakutskiya
26	*Ephedra przewalskii*	China North-Central, Inner Mongolia, Kazakhstan, Kirgizstan, Mongolia, Pakistan, Qinghai, Tadzhikistan, Uzbekistan, Xinjiang
27	*Ephedra sinica*	Buryatiya, China North-Central, Qinghai Chita, Inner Mongolia, Manchuria, Mongolia, Primorye
28	*Ginkgo biloba*	China Southeast, Qinghai
29	*Gnetum pendulum*	China South-Central, China Southeast, Tibet
30	*Abies chayuensis*	China South-Central, Tibet
31	*Abies delavayi var. delavayi*	China South-Central, East Himalaya, Myanmar, Tibet, Vietnam
32	*Abies delavayi var. motuoensis*	Tibet
33	*Abies densa*	Assam, East Himalaya, Nepal, Tibet
34	*Abies ernestii*	China North-Central, China South-Central, Qinghai, Tibet
35	*Abies ernestii var. salouenensis*	China South-Central, East Himalaya, Tibet
36	*Abies fargesii*	China North-Central, China South-Central, Qinghai
37	*Abies fargesii var. faxoniana*	China South-Central, Qinghai
38	*Abies forrestii*	China South-Central, Tibet
39	*Abies georgei*	China South-Central, Tibet
40	*Abies georgei var. smithii*	China South-Central, Tibet
41	*Abies spectabilis*	Afghanistan, Nepal, Pakistan, Tibet, West Himalaya
42	*Abies squamata*	China North-Central, China South-Central, Qinghai, Tibet
43	*Larix gmelinii*	Amur, Buryatiya, China North-Central, China Southeast, Qinghai, Chita, Inner Mongolia, Irkutsk, Kamchatka, Khabarovsk, Korea, Kuril Is., Magadan, Manchuria, Mongolia, Primorye, Sakhalin, Yakutskiya
44	*Larix griffithii*	China South-Central, East Himalaya, Nepal, Tibet
45	*Larix himalaica*	Nepal, Tibet
46	*Larix kaempferi*	Qinghai, Japan
47	*Larix olgensis*	Qinghai, Korea, Manchuria, Primorye
48	*Larix potaninii*	China North-Central, China South-Central, Nepal, Qinghai, Tibet

49	*Larix potaninii var. australis*	China North-Central, China South-Central, Tibet
50	*Larix gmelinii var. principis-rupprechtii*	China North-Central, China Southeast, Inner Mongolia, Qinghai
51	*Larix speciosa*	China South-Central, Tibet
52	*Picea brachytyla*	China North-Central, China South-Central
53	*Picea brachytyla var. complanata*	Tibet, China Southwest
54	*Picea crassifolia*	China North-Central, Inner Mongolia, Qinghai
55	*Picea likiangensis var. rubescens*	China South-Central, Qinghai, Tibet
56	*Picea likiangensis var. hirtella*	China South-Central, Tibet
57	*Picea likiangensis var. linzhiensis*	China South-Central, Tibet
58	*Picea purpurea*	China North-Central, China South-Central, Qinghai
59	*Picea asperata*	China North-Central, China South-Central, Inner Mongolia, Qinghai
60	*Picea schrenkiana*	Kazakhstan, Kirgizstan, Xinjiang, Tibet
61	*Picea smithiana*	Afghanistan, Nepal, Pakistan, Tibet, West Himalaya
62	*Picea spinulosa*	East Himalaya, Tibet
63	*Picea wilsonii*	China North-Central, China South-Central, Inner Mongolia, Qinghai
64	*Pinus armandii*	China North-Central, China South-Central, China Southeast, Hainan, Inner Mongolia, Myanmar, Taiwan, Tibet
65	*Pinus bhutanica*	China South-Central, East Himalaya, Tibet
66	*Pinus densata*	China South-Central, Tibet
67	*Pinus gerardiana*	Afghanistan, Pakistan, Tibet, West Himalaya
68	*Pinus wallichiana*	Afghanistan, China South-Central, East Himalaya, Nepal, Pakistan, Tibet, West Himalaya
69	*Pinus roxburghii*	Bangladesh, East Himalaya, India, Nepal, Pakistan, West Himalaya, Tibet
70	*Pinus sylvestris var. mongolica*	Buryatiya, Chita, Inner Mongolia, Irkutsk, Manchuria, Mongolia
71	*Pinus tabuliformis*	China North-Central, China South-Central, China Southeast, Inner Mongolia, Korea, Manchuria, Qinghai
72	*Pinus yunnanensis*	China South-Central, China Southeast, Tibet
73	*Pseudotsuga forrestii*	China North-Central, China South-Central, China Southeast, Tibet, Taiwan
74	*Tsuga dumosa*	China South-Central, East Himalaya, Myanmar, Nepal, Tibet, West Himalaya
75	*Podocarpus neriifolius*	Andaman Is., Assam, Bangladesh, Borneo, Cambodia, Malaya, Myanmar, Tibet, Nepal, Philippines, Sulawesi, Sumatera, Thailand, Vietnam
76	*Cephalotaxus mannii*	Assam, China South-Central, China Southeast, East Himalaya, Laos, Myanmar, Thailand, Tibet, Vietnam
77	*Amentotaxus argotaenia*	Cambodia, China North-Central, China South-Central, China Southeast, Laos, Tibet, Vietnam
78	*Amentotaxus assamica*	East Himalaya, Tibet

79	*Taxus wallichiana*	China South-Central, East Himalaya, Laos, Myanmar, Nepal, Philippines, Sulawesi, Sumatera, Tibet, Vietnam

Table S2. Pearson correlation coefficient shows the correlation between climatic conditions, and species richness.

	Elevation	SR	GDD5	GDD0	MAT	MI	DI	GP	MAP	SS%	Tmax	Tmin	MTCO	MTWA	PDM	PWM	TAR	MDT	PS	TS
**Elevation**		0.042	0.000	0.000	0.000	0.003	0.410	0.000	0.000	0.000	0.000	0.000	0.000	0.000	0.000	0.365	0.000	0.000	0.000	0.000
**SR**	0.281		0.001	0.005	0.064	0.000	0.000	0.146	0.209	0.279	0.012	0.542	0.219	0.015	0.120	0.000	0.001	0.124	0.887	0.002
**GDD5**	-0.977	-0.451		0.000	0.000	0.099	0.637	0.000	0.000	0.000	0.000	0.000	0.000	0.000	0.000	0.713	0.000	0.000	0.000	0.000
**GDD0**	-0.993	-0.380	0.995		0.000	0.022	0.892	0.000	0.000	0.000	0.000	0.000	0.000	0.000	0.000	0.825	0.000	0.000	0.000	0.000
**MAT**	-0.997	-0.256	0.973	0.989		0.003	0.403	0.000	0.000	0.000	0.000	0.000	0.000	0.000	0.000	0.282	0.000	0.000	0.000	0.000
**MI**	-0.406	0.523	0.229	0.313	0.407		0.000	0.001	0.000	0.000	0.018	0.000	0.000	0.013	0.000	0.000	0.000	0.000	0.000	0.000
**DI**	0.115	-0.618	0.066	-0.019	-0.117	-0.951		0.193	0.000	0.000	0.821	0.032	0.126	0.755	0.018	0.000	0.000	0.000	0.001	0.000
**GP**	-0.983	-0.203	0.943	0.968	0.988	0.459	-0.182		0.000	0.000	0.000	0.000	0.000	0.000	0.000	0.106	0.000	0.000	0.000	0.000
**MAP**	-0.812	0.175	0.681	0.746	0.807	0.855	-0.662	0.837		0.000	0.000	0.000	0.000	0.000	0.000	0.000	0.000	0.000	0.000	0.000
**SS%**	0.859	-0.151	-0.738	-0.795	-0.851	-0.782	0.558	-0.862	-0.982		0.000	0.000	0.000	0.000	0.000	0.000	0.000	0.000	0.000	0.000
**Tmax**	-0.991	-0.343	0.982	0.991	0.984	0.324	-0.032	0.971	0.756	-0.810		0.000	0.000	0.000	0.000	0.702	0.000	0.000	0.000	0.000
**Tmin**	-0.976	-0.086	0.912	0.945	0.979	0.567	-0.295	0.980	0.899	-0.930	0.953		0.000	0.000	0.000	0.016	0.000	0.000	0.000	0.000
**MTCO**	-0.991	-0.172	0.947	0.972	0.994	0.494	-0.213	0.990	0.861	-0.897	0.975	0.995		0.000	0.000	0.074	0.000	0.000	0.000	0.000
**MTWA**	-0.997	-0.333	0.988	0.997	0.995	0.338	-0.044	0.979	0.766	-0.816	0.995	0.960	0.982		0.000	0.637	0.000	0.000	0.000	0.000
**PDM**	-0.936	-0.216	0.882	0.913	0.922	0.576	-0.325	0.916	0.892	-0.904	0.906	0.934	0.934	0.915		0.099	0.000	0.000	0.000	0.000
**PWM**	-0.127	0.732	-0.052	0.031	0.150	0.880	-0.913	0.225	0.616	-0.526	0.054	0.329	0.247	0.066	0.229		0.000	0.000	0.003	0.000
**TAR**	0.670	-0.445	-0.514	-0.587	-0.686	-0.889	-0.737	-0.728	-0.931	0.906	-0.608	-0.814	-0.757	-0.621	-0.718	-0.804		0.000	0.000	0.000

**MDT**	0.848	-0.214	-0.730	-0.787	-0.862	-0.775	0.557	-0.880	-0.957	0.960	-0.791	-0.940	-0.905	-0.813	-0.860	-0.601	0.952		0.000	0.000
**PS**	0.930	-0.020	-0.835	-0.883	-0.925	-0.679	0.425	-0.936	-0.955	0.977	-0.891	-0.973	-0.955	-0.900	-0.950	-0.399	0.854	0.954		0.000
**TS**	0.689	-0.421	-0.538	-0.609	-0.707	-0.879	0.722	-0.746	-0.934	0.910	-0.625	-0.828	-0.774	-0.642	-0.730	-0.790	0.999	0.962	0.863	

Table S3. Loading of environmental variables in PCA analysis

Environmental variables	PC 1	PC 2
GDD5	**0.24512**	0.092666
GDD0	**0.24797**	0.12184
MAT	**0.23895**	0.20233
MI	**0.22042**	-0.25906
DI	-0.18591	**0.35743**
GP	**0.2395**	0.075439
MAP	**0.25227**	-0.06391
SS%	-0.25033	**0.009061**
Tmax	0.22566	**0.2734**
Tmin	**0.25041**	0.10046
MTCO	**0.24572**	0.15114
MTWA	0.2292	**0.25813**
PDM	**0.2317**	0.16169
PWM	**0.17458**	-0.41061
TAR	-0.23921	**0.19665**
MDT	-0.2517	**0.079393**
PS	-0.24993	**-0.05837**
TS	-0.24039	**0.18741**
SR	**0.054668**	-0.52759
Eigen value	15.4319	2.76236
% Variance	81.221	14.539
Significant	**0.0001**	0.05

Table S4. Percentage of coefficient of determination (R^2_{adj}, %) by the generalized linear models (GLMs) between species richness of gymnosperms and predictor variables sets representing each hypothesis set. *p*-value is the significance value of each model. Numbers in parentheses are the coefficient of respective variables.

Environmental predictor sets	Variables	Coeff.	Std. error	t	F	p	$R^2_{adj\ (\%)}$
**Energy-Water**	GDD_5	-42.920	18.877	-2.274	5.169	0.028	10.1
	GDD_0	-38.616	23.566	-1.639	2.685	0.108	5.5
	MAT	-0.048	0.080	-0.594	0.352	0.556	0.8
	MI	0.001	0.000	3.726	13.880	**0.001**	23.2
	DI	-0.001	0.000	-4.503	20.270	**>0.000**	30.6
	GP	-0.908	2.749	-0.330	0.109	0.743	0.2
	MAP	2.282	1.443	1.582	2.502	0.121	5.2
	SS%	-0.024	0.014	-1.691	2.858	0.098	5.9
**Physical tolerance**	T_{max}	-0.095	0.071	-1.349	1.820	0.184	3.8
	T_{min}	0.072	0.138	0.525	0.275	0.602	0.6
	MTCO	-0.004	0.088	-0.041	0.002	0.968	0.0
	MTWA	-0.083	0.068	-1.219	1.486	0.229	3.1
	PDM	-0.011	0.016	-0.692	0.479	0.492	1.0
	PWM	0.592	0.091	6.510	42.380	**0.000**	48.0
**Climatic seasonality**	TAR	-0.080	0.020	-3.884	15.090	**0.000**	24.7
	MDT	-0.014	0.006	-2.440	5.954	**0.019**	11.5
	Precipitation Seasonality	-0.207	0.207	-1.001	1.003	0.322	2.1
	Temperature Seasonality	-0.027	0.007	-3.716	13.800	**0.001**	23.1
